# Overcoming the Challenge: A Comprehensive Review of Neoadjuvant Treatment Resistance in Rectal Cancer

**DOI:** 10.1007/s12029-025-01324-7

**Published:** 2025-10-15

**Authors:** Alexandru Micu, Andrei Diaconescu, Corina-Elena Minciuna, Teodora Manuc, Simona Olimpia Dima, Gabriela Droc, Vlad Herlea, Gabriel Becheanu, Adina Emilia Croitoru, Catalin Vasilescu

**Affiliations:** 1 Department of General Surgery, Fundeni Clinical Institute, Bucharest, Romania; 2 “Carol Davila” University of Medicine and Pharmacy, Bucharest, Romania; 3 Department of Gastroenterology and Hepatology, Fundeni Clinical Institute, Bucharest, Romania; 4 Centre of Anaesthesia and Intensive Care, Fundeni Clinical Institute, Bucharest, Romania; 5 Department of Pathology, Fundeni Clinical Institute, Bucharest, Romania; 6 Department of Oncology, Fundeni Clinical Institute, Bucharest, Romania; 7 Center of Excellence in Translational Medicine (CEMT), Fundeni Clinical Institute, Bucharest, Romania

**Keywords:** Rectal cancer, Oncology, Treatment resistance, Neoadjuvant therapy, Response assessment

## Abstract

Colorectal cancer (CRC) is the third most commonly diagnosed cancer and remains a leading cause of cancer-related mortality, particularly among younger men. Approximately one-third of colorectal cancers occur in the rectum. For patients with locally advanced rectal cancer, neoadjuvant therapy is considered the standard treatment approach. Despite advances in therapeutic approaches, improvements in the 5-year survival rate have been modest. Accurate assessment of tumor response to neoadjuvant therapy (NAT) is critical for guiding subsequent treatment strategies, especially when considering eligibility for non-operative management (NOM). Common evaluation methods include digital rectal examination (DRE), magnetic resonance imaging (MRI), and high-definition flexible endoscopy (HDFE). Tumor regression grading (TRG) systems—both histopathological (pTRG) and MRI-based (mrTRG)—are valuable tools for quantifying treatment response and predicting long-term outcomes. However, resistance to NAT remains a significant clinical challenge and is driven by a complex interplay of molecular mechanisms. Genetic factors, such as RAS mutations, have been linked to resistance to chemoradiotherapy (CRT), while tumors exhibiting microsatellite instability (MSI-high) tend to respond poorly to CRT but may show favorable outcomes with immune checkpoint inhibitors. Epigenetic pathways, including dysregulation of Wnt/β-catenin and PI3K/AKT signaling, along with alterations in DNA damage repair mechanisms, further influence CRT sensitivity. The tumor microenvironment also plays a pivotal role in modulating therapy response. Elements such as immune cell infiltration, hypoxia, angiogenesis, and the presence of cancer-associated fibroblasts (CAFs) contribute to a pro-resistance landscape. Moreover, emerging evidence suggests that gut microbiota composition—particularly an enrichment of *Bacteroides* species—is associated with diminished response to NAT. Understanding these multifaceted biological interactions is essential for developing personalized and more effective therapeutic strategies, with the goal of enhancing response to NAT and ultimately improving clinical outcomes in patients with rectal cancer.

## Introduction

Colorectal cancer (CRC) ranks as the third most commonly diagnosed cancer and the third leading cause of cancer-related deaths in both men and women in the USA. Notably, it is the leading cause of cancer-related deaths among men under the age of 50 [1]. Approximately one-third of CRC cases occur in the rectum, with an estimated 46,220 new cases of rectal cancer expected in the USA in 2024 [1]. Despite advancements in treatment strategies, the 5-year survival rate for rectal cancer has shown only modest improvement, rising from 62% in 1995–1997 to 67% in 2013–2019 [2].

The standard multimodal treatment for locally advanced rectal cancer (LARC) consists of neoadjuvant chemoradiotherapy (NACRT), followed by total mesorectal excision (TME) surgery and adjuvant chemotherapy [3]. Neoadjuvant treatment (NAT) with 5-fluorouracil (5-FU) remains the standard of care in the USA and Europe due to its effectiveness in tumor downstaging, achieving higher rates of pathological complete response (pCR), reducing local recurrence, and improving quality of life by increasing the likelihood of sphincter-sparing surgeries [4]. Because of this, and to maintain a focused and clinically relevant scope, the present review addresses mechanisms of resistance exclusively in the neoadjuvant setting, where resistance poses the greatest therapeutic challenge and has the most direct implications for current patient care.

In recent years, total neoadjuvant therapy (TNT) has gained popularity in rectal cancer management. TNT involves combining systemic chemotherapy—such as CAPOX or FOLFOX—alongside chemoradiotherapy (CRT) with capecitabine or 5-FU before radical surgery [5]. Studies have demonstrated that TNT significantly improves disease-free survival (DFS), metastasis-free survival (MFS), and local control, with pCR rates nearly doubling. Consequently, recent guidelines now recommend TNT for stage II–III rectal cancers [1, 6].

Over half of CRC patients will develop metastases, typically metachronously following treatment for locoregional disease. The liver is the most common site of metastasis, with 80–90% of patients being inoperable at diagnosis [7]. This highlights the need for more effective systemic therapies and earlier detection strategies to improve long-term outcomes.

Immunotherapy, particularly immune checkpoint inhibitors, has shown considerable promise. Anti-PD-1 therapies have demonstrated high efficacy in microsatellite instability-high (MSI-H) or mismatch repair-deficient (dMMR) advanced CRC, with some studies reporting potentially curative outcomes due to high pathological complete response (pCR) rates [8]. Although immunotherapy has not yielded significant benefits in proficient mismatch repair/microsatellite-stable (pMMR/MSS) tumors, emerging evidence suggests that combining neoadjuvant immunotherapy with CRT may offer benefits even in this subgroup [9].

Over two decades ago, the concept of non-operative management (NOM) emerged for rectal cancer. This approach, known as “Watch and Wait” (WW), involves close surveillance in patients who achieve a complete response following neoadjuvant therapy [10]. Complete clinical response (cCR) is determined through digital rectal examination, magnetic resonance imaging (MRI), and flexible endoscopy. If no residual tumor is detected across these evaluations (see Table 1), patients may be considered for the WW approach [11].

**Table 1:** The classification of treatment responses in rectal cancer following neoadjuvant therapy is based on a comprehensive assessment combining digital rectal examination (DRE), diffusion-weighted magnetic resonance imaging (MRI), and high-definition flexible endoscopic evaluations (HDFE), supplemented by histopathological examination (HP) when applicable, ensuring a comprehensive and accurate assessment of treatment efficacy, guiding subsequent treatment decisions [18, 21–23]

Type of response	HDFE	DRE	MRI	Assessment
Complete clinical response (*cCR*)	Pale smooth scar ± telangiectasia	Smooth, flat scar	T2 weighted—fibrotic, linear scar with low signal intensity	All criteria must be satisfied
	No ulceration, nodularity, or mucosal irregularities	No nodularity	Only dark T2 signal	Induction CHT followed by RT—> evaluation no earlier than 8 weeks
	No stricture		No suspicious lymph nodes	RT followed by consolidation CHT—> evaluation within a month
Near complete clinical response (*nCR*)	Irregular small mucosal nodules, superficial ulceration or mild persistent erythema	Smooth induration or superficial minor mucosal irregularity	T2 weighted—downstaging ± residual fibrosis, small area of residual signal, complete/partial regression of lymph nodes	If patient wishes to avoid surgery—additional 8 weeks of observation followed by reassessment
			Mostly dark T2 signal	
			Small area of residual high signal intensity	
Incomplete/partial clinical response (iCR)	Visible tumor	Palpable tumor nodule	T2 weighted-more intermediate than a dark T2 signal, no T2 scar +/no regression of lymph nodes	

Patients with dMMR or MSI-H tumors who achieve cCR after immune checkpoint inhibitor therapy may also be suitable candidates for NOM. Trials have shown that all stage II and III dMMR rectal adenocarcinoma patients who received anti-PD-1 immunotherapy and completed treatment maintained cCR during follow-up [1, 8].

A significant proportion of rectal cancers exhibit resistance to neoadjuvant therapies. This resistance is characterized by poor tumor downstaging, low rates of pCR, early recurrence, metastasis, and limited long-term survival.

The *objective* of this review is to evaluate the current literature to identify factors contributing to resistance to neoadjuvant treatment in rectal cancer. Additionally, it aims to determine prognostic and predictive factors that can guide personalized treatment strategies for improved patient outcomes.

## How We Define Cancer Resistance to Neoadjuvant Treatment?

Cancer resistance to treatment is defined by the capacity of malignant cells to evade the cytotoxic effects of therapies, reducing treatment effectiveness through a complex array of mechanisms that enable these cells to survive even the most aggressive therapeutic approaches [12].

Radiotherapy resistance is divided into two categories: intrinsic resistance (this refers to pre-existing traits of cancer cells that make them less sensitive to radiation before treatment begins) and acquired resistance (develops during or after treatment, often influenced by the protective effect of the tumoral microenvironment) [13].

**Fig. 1:**
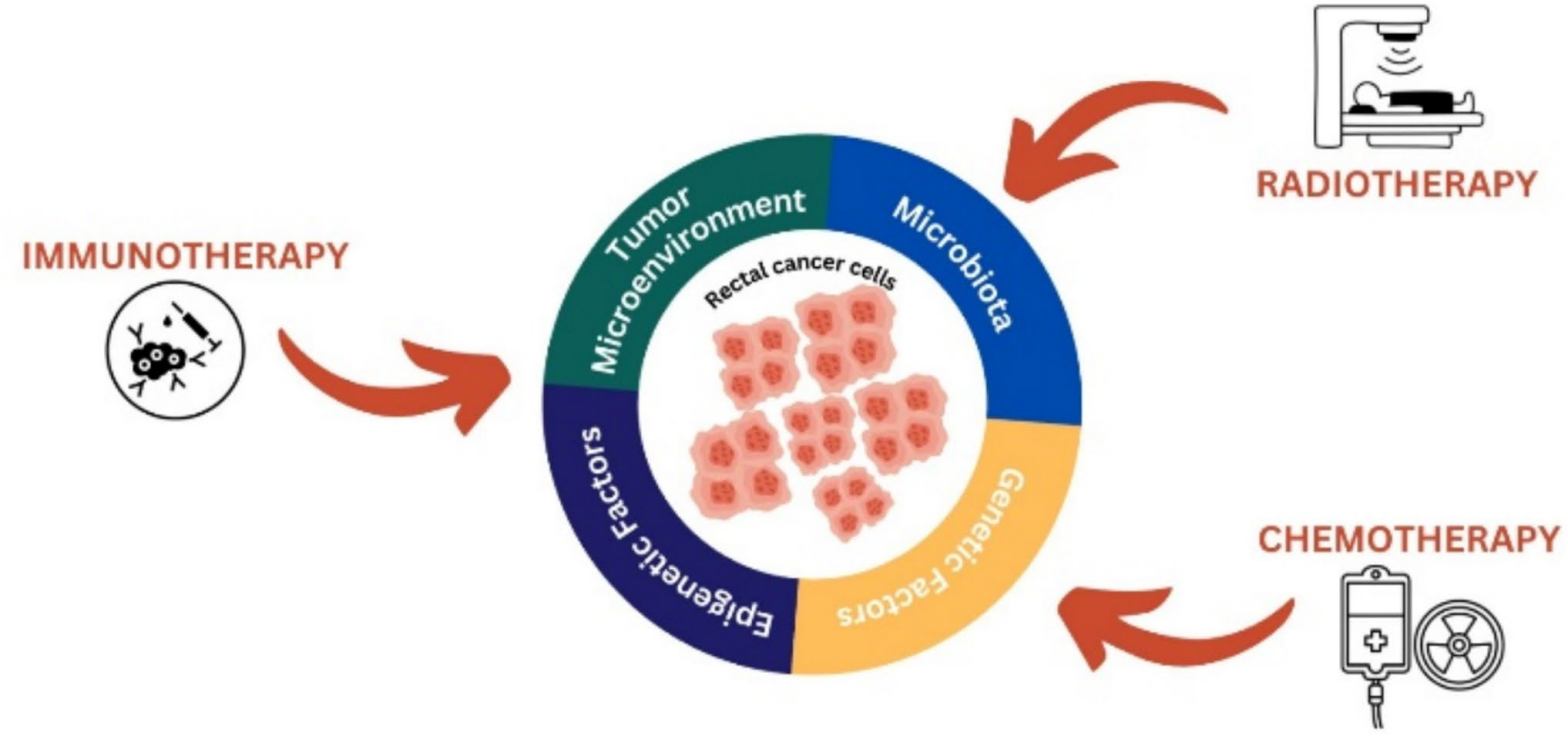
Multimodal influences on rectal cancer cells. This schematic illustrates the multifactorial influences on rectal cancer cells, including the tumor microenvironment, microbiota, genetic factors, and epigenetic factors. These internal and external components shape tumor behavior and response to treatment. Surrounding this central core, three major therapeutic modalities—immunotherapy, chemotherapy, and radiotherapy—are shown as external interventions targeting rectal cancer. The figure highlights the interplay between tumor biology and treatment strategies in a comprehensive approach to rectal cancer management

Chemotherapy resistance is divided into three categories: inherent, acquired, and adaptative resistance. Inherent resistance is primarily attributed to the genetic and epigenetic landscape of cancer cells, which predisposes certain tumors to exhibit insensitivity to conventional therapies[14]. In contrast, acquired or extrinsic resistance arises under treatment, promoting the expansion of resistant subclones through multiple adaptative responses within the tumor that was at first sensitive to treatment [15]. Moreover, adaptive resistance arises when cancer cells rapidly reprogram their signalling pathways, gene expression, and cellular functions in real time in response to therapeutic pressure; this dynamic adaptation can diminish the efficacy of treatment, even in the absence of stable genetic alterations [12]. Figure 1 provides a schematic overview of the multifaceted factors influencing rectal cancer biology and therapeutic response, illustrating the complex interplay between tumor-intrinsic mechanisms and external treatment modalities.

## Assessment of Response in Rectal Cancer Following Neoadjuvant Therapy

Assessing the response to NAT is a critical step in managing rectal cancer, influencing decisions regarding surgical planning and the potential for non-operative management (NOM). Achieving a complete clinical response (cCR) is significant, as it can guide treatment strategies, including the possibility of NOM, and also impacts surgical approaches by accounting for tumor-induced anatomical changes following NAT [16].

The primary tools for evaluating cCR include digital rectal examination (DRE), magnetic resonance imaging (MRI), and high-definition flexible endoscopy (HDFE). Each modality has specific criteria to assess tumor presence or absence after NAT (see Table 1). Identification of a cCR can support NOM, which is especially beneficial for patients with low rectal tumors where surgery might involve abdominoperineal resection or very low anterior resection with colo-anal anastomosis—both of which significantly affect quality of life (QOL) [17].

Patients who do not achieve cCR initially, but exhibit substantial tumor regression, are classified as near-complete responders (nCR) (see Table 1). For these patients, current guidelines recommend a reassessment after an additional 8 weeks of observation, as some may progress to cCR within this interval [11, 18]. Moreover, recent research suggests incorporating lymph node status in defining nCR. In organ-preserving strategies, locoregional lymph nodes remain intact, and the presence of metastatic nodes is associated with an increased risk of local recurrence [19, 20].

This structured and comprehensive assessment approach ensures accurate clinical decision-making, optimizing treatment outcomes for rectal cancer patients undergoing NAT.

Accurate evaluation of response to neoadjuvant therapy (NAT) in rectal cancer is critical for guiding subsequent treatment strategies and improving patient outcomes. However, magnetic resonance imaging (MRI) and endoscopy demonstrate limited sensitivity in detecting complete clinical response (cCR), and endoscopic biopsies often yield inaccurate results. To overcome these limitations, recent studies highlight the effectiveness of probe-based confocal laser endomicroscopy (pCLE) in evaluating response to NAT and monitoring sustained responses during the “Watch and Wait” (WW) approach [24, 25]. pCLE is a real-time, in vivo imaging technique that provides optical biopsies with × 100 magnification, allowing detailed evaluation of cellular and vascular patterns.

Pathological complete response (pCR) is confirmed through histopathological examination and is defined as ypT0N0 at the time of surgery (see Table 1) [21]. Nearly 40% of patients receiving total neoadjuvant treatment (TNT) can achieve pCR, which is associated with excellent prognostic outcomes, including a 5-year survival rate of 95% [26].

A widely used method to evaluate tumor response following neoadjuvant chemoradiotherapy (NACRT) is the tumor regression grade (TRG). TRG categorizes patients based on the extent of histological changes in the surgical specimen, specifically assessing the proportion of the tumor mass replaced by fibrosis [27]. Various TRG systems are in use, but recent studies advocate for adopting the American Joint Committee on Cancer (AJCC) system (Table 2). This recommendation is based on the routine application of AJCC’s TNM staging, its ability to ensure uniform data interpretation, and its critical role in predicting prognosis and guiding decisions regarding adjuvant chemotherapy [27, 28].

**Table 2:** Magnetic resonance tumor regression grade (mrTRG)

mrTRG	Type of response	Response description
Grade 1 (complete radiologic regression)	Complete response	No evidence of treated tumor
Grade 2 (good regression)	Near-complete response	Extensive fibrosis (> 75%) with minimal or no remaining tumor, indicating minimal residual disease or absence of tumor
Grade 3 (moderate regression)	Partial response	Over 50% fibrosis or mucin and visible intermediate signal intensity
Grade 4 (slight regression)	Poor response	Small areas of fibrosis or mucin, but predominantly tumor
Grade 5 (no regression)	No response	Exhibiting the same appearance as the original tumor

This comprehensive and advanced approach enhances the accuracy of response assessment and informs tailored treatment strategies for rectal cancer patients undergoing NAT.

Given the high resolution of magnetic resonance imaging (MRI) and its ability to differentiate between residual cancer and fibrosis, an MRI-based tumor regression grade (mrTRG) system was developed [29]. Unlike pathological TRG (pTRG), which is determined postoperatively, mrTRG provides a preoperative, non-invasive assessment of tumor response to neoadjuvant therapy.

The mrTRG system plays a crucial role in the decision-making process, allowing for early stratification of treatment strategies. Accurate imaging-based classification can guide the adjustment of therapies to enhance response or support the adoption of a non-operative approach in cases of complete response [29, 30].

This system enhances the ability to personalize treatment pathways, optimizing outcomes and minimizing unnecessary surgical interventions.

## Chemoradiotherapy Resistance in Rectal Cancer

### Genetic Factors

Genetic profiling plays a crucial role in characterizing rectal tumors, as specific mutations serve as predictive and prognostic biomarkers for treatment response. Key genetic factors, such as RAS mutations and microsatellite instability (MSI), significantly influence the effectiveness of NAT and overall treatment outcomes.

#### RAS Mutations

RAS status testing, particularly for KRAS, is essential in predicting treatment response in rectal cancer [31]. Several studies have highlighted the impact of RAS mutations on chemoradiotherapy (CRT) resistance. Bedrikovetski et al*.* analyzed 80 patients with advanced rectal cancer and found that those with RAS/BRAF mutations had significantly lower rates of cCR and oCRr, although no significant difference was observed in pathological complete response (pCR) rates [32]. Similarly, Bahnassy et al*.* demonstrated a significant association between KRAS mutations and poor treatment outcomes, reporting that only 4 out of 25 KRAS-mutant patients achieved pCR [33]. Another study found that one-third of LARC patients with RAS mutations did not respond to NAT, with lower tumor regression grades compared to wild-type patients [34].

In contrast, a large retrospective analysis by Zhou et al*.*, which included 1886 stage II and III rectal cancer patients, showed no significant association between mutant KRAS (mKRAS) status and pCR or low neoadjuvant rectal (NAR) scores. The study concluded that KRAS mutation did not impact tumor downstaging after NAT [35]. However, another study involving 381 patients indicated that KRAS wild-type status and younger age were independent predictors for achieving pCR [36].

#### Microsatellite Instability (MSI)

MSI arises from the loss of mismatch repair (MMR) proteins, including MLH1, MSH2, MSH6, and PMS2 [37]. Tumors characterized as MSI-high (MSI-H) exhibit instability in over 40% of tested microsatellite loci [38]. MSI testing is recommended for all rectal cancer patients due to its role in predicting chemoresistance and prognosis [39–41].

Studies have consistently shown that deficient MMR (dMMR) tumors are associated with lower pCR rates, highlighting the importance of evaluating MMR status, particularly when considering non-operative approaches [42]. Farchoukh et al*.* revealed that tumor downstaging after NAT was significantly lower in MMR-deficient (dMMR) patients (11%) compared to MMR-proficient (pMMR) patients (57%, *p* = 0.007). Additionally, dMMR was associated with poor response (TRG-3) in multivariable logistic regression analysis [43].

Hasan et al*.* also reported that MSI-positive tumors were independently linked to reduced pCR rates. This finding supports MSI testing, especially when considering non-operative management for patients requiring abdominoperineal resection, as MSI-positive tumors may demonstrate relative resistance to CRT [44]. Another study, conducted on 318 LARC patients, showed that dMMR/MSI-H tumors exhibited a lower response to standard CRT but demonstrated excellent responses to immune checkpoint inhibitors. This suggests that dMMR/MSI-H rectal cancers represent a distinct disease entity compared to pMMR/MSS tumors [45]. In their cohort, 42.6% of pMMR patients exhibited major TRG (TRG-0 or TRG-1), while none of the dMMR patients reached a major TRG [45]. However, it is important to note that some studies have reported no significant association between MMR status and tumor response to NAT in LARC patients [46].

Although current evidence indicates a strong association between RAS mutations, MSI status, and resistance to chemoradiotherapy in rectal cancer, the findings remain heterogeneous and inconclusive across studies. Further research is required to fully understand these genetic influences and develop more personalized treatment strategies to optimize outcomes for rectal cancer patients.

### Epigenetic Factors

Multiple molecular pathways, including Wnt/β-catenin, PI3K/AKT, and DNA damage repair, contribute to chemoradiotherapy resistance in rectal cancer. Enzymes, proteins, and specific gene expressions further influence tumor response to treatment. Understanding these mechanisms is crucial for developing targeted therapeutic strategies aimed at overcoming resistance and improving patient outcomes.

#### Wnt/β-Catenin Pathway

The Wnt/β-catenin signalling pathway is essential for embryonic development and tissue homeostasis. Aberrations in this pathway contribute to the development and progression of various cancers, including colorectal cancer, with over 90% of CRC cases showing alterations in Wnt signalling [47].

Miyako et al*.* demonstrated that high nuclear β-catenin expression is significantly associated with poor response to neoadjuvant chemoradiotherapy. While nuclear β-catenin accumulation was linked to resistance through the regulation of cancer stem cells (CSCs) and epithelial-mesenchymal transition (EMT), the study found no association with CD44, a known CSC marker. This suggests that Wnt/β-catenin signalling may influence NACRT response primarily via EMT modulation [48, 49].

Wen et al*.* identified that destrin (DSTN), an actin depolymerizing factor, was highly expressed and hypomethylated in rectal cancer tissues resistant to radiation therapy. Inhibition of DNA methylation resulted in increased DSTN expression. Moreover, tumors overexpressing DSTN showed activation of the Wnt/β-catenin pathway, with both β-catenin and DSTN highly expressed in radiation-resistant tissues [50].

#### PI3K/AKT Pathway

The PI3K/AKT/mTOR signalling pathway plays a crucial role in cell proliferation and survival, contributing to CRT resistance. Wanigasooriya et al*.* used patient-derived primary cell culture models to investigate resistance mechanisms, revealing that upregulation of PI3K/AKT/mTOR was a key factor. Their study also demonstrated that dual PI3K/mTOR inhibitors could enhance radiotherapy effectiveness in resistant patient-derived organoids (PDOs) [51].

Ferrandon et al*.* highlighted the role of coenzyme A synthase (CASY) in promoting cell survival and enhancing DNA damage repair after radiation. CASY augments radiation-induced PI3K/AKT activation, increasing p-AKT and p-mTOR levels while also facilitating DNA damage repair, contributing to resistance [52].

Tian et al*.* identified the potassium voltage-gated channel subfamily E regulatory subunit 4 (KCNE4) as a promoter of radiation resistance. KCNE4 enhances cancer cell viability and inhibits apoptosis by activating the PI3K/AKT pathway, correlating with poor survival and increased immune cell infiltration in CRC [53].

#### DNA Damage Repair (DDR)

Efficient DNA damage repair mechanisms significantly contribute to radiation resistance. Wei et al*.* reported that CRC cells with mitochondrial dysfunction exhibited increased radiation resistance and enhanced DNA damage repair capabilities [54]. Elevated expression of DDR-related genes at both transcriptomic and protein levels was observed, indicating an improved DNA repair response [54].

PRDM15, a zinc-finger protein, was identified as a critical factor in regulating non-homologous end joining (NHEJ)–mediated DDR [55]. Knockdown of PRDM15 impaired DNA repair and increased radiosensitivity in rectal cancer cells [55].

Wang et al*.* found that replication factor C subunit 4 (RFC4) promotes NHEJ-mediated DNA repair by interacting with Ku70/Ku80 [56]. RFC4 upregulation was associated with reduced tumor regression and poor prognosis in LARC patients undergoing NAT [56].

Overexpression of ERCC1 was also linked to CRT resistance, likely due to its role in enhancing DNA repair capacity and increasing tolerance to radiation [57].

#### Other Epigenetic Factors

High expression of serine peptidase inhibitor Kazal type-1 (SPINK1) is associated with poor prognosis, advanced cancer stages, and increased metastasis in CRC [58, 59]. Chen et al*.* demonstrated that high SPINK1 expression, both before and after CRT, correlated with perineural invasion and poor treatment response [60].

RIO kinase 1 was identified as a negative prognostic factor, with its overexpression suppressing p53 signalling and reducing tumor regression after NAT [61].

Zhou et *al.* reported that overexpression of serum and glucocorticoid-regulated kinase 1 (SGK1) reduced radiosensitivity in rectal cancer cells by activating transcription factor 3 (ATF3) [62]. Downregulating SGK1 or ATF3 enhanced the radiation response [62].

Souza e Silva et al*.* assessed thymidylate synthase (TYMS) and RAD23 homolog B (RAD23B) in circulating tumor cells (CTCs) before and after CRT [63]. The absence of RAD23B after CRT correlated with pCR, highlighting its potential as a predictive marker for treatment response [63].

Nuclear factor erythroid 2-related factor 2 (Nrf2) is involved in oxidative stress regulation [64]. High Nrf2 expression was associated with poor therapeutic response in LARC patients [65].

Kubota et al*.* identified sulphur metabolism-related proteins as significant discriminators in CRT-resistant tumors [66]. Selenium-binding protein 1 (SELENBP1) expression was reduced in resistant cases, correlating with lower NAC sensitivity [66].

S100A4, a calcium-binding protein, was shown to promote CRT resistance by inhibiting p53 activation and DNA binding, contributing to tumor survival [67]. Wild-type p53 cells with low S100A4 expression were more sensitive to CRT-induced apoptosis [67].

Huang et al*.* identified zinc finger protein 37 A (ZNF37A) as significantly correlated with CRT resistance [68]. High ZNF37A expression enhanced sensitivity to NAT by promoting apoptosis through the inhibition of TNFRSF6B transcription [68].

BEAN1 (Brain Expressed Associated with NEDD4-1) is a brain-expressed protein linked to NEDD4-1, an E3 ubiquitin ligase involved in neural development and synaptic function with a critical role in spinocerebellar ataxia type 31 [69]. Shapaer et al*.* reported that BEAN1 promoted resistance and tumor progression by modulating Wnt/β-catenin signalling and enhancing EMT through TGF-β signalling [70].

### Tumor Microenvironment

The tumor microenvironment (TME) is a complex ecosystem composed of tumor cells, stromal cells (primarily fibroblasts), immune cells, and non-cellular components of the extracellular matrix. These components collectively influence tumor initiation, progression, invasion, metastasis, and response to therapies [71]. The tumor microenvironment significantly influences chemoradiotherapy resistance in rectal cancer. Immune cells, hypoxia-induced metabolic changes, angiogenesis, and cancer-associated fibroblasts contribute to the complex interplay that determines treatment outcomes. Understanding these mechanisms is essential for developing targeted therapies aimed at overcoming resistance and improving clinical outcomes.

#### Immune Cells and Tumor Resistance

Immune cells play a pivotal role in anti-tumor responses. Chronic inflammation promotes immune cell aggregation, particularly in non-lymphoid tissues at pathological sites. Tertiary lymphoid structures (TLSs), clusters of immune cells attached to tumor tissues, enhance systemic immune responses and are a primary source of tumor-infiltrating lymphocytes (TILs) [72].

Zhang et al*.* demonstrated that the expression levels of CD4 + and CD8 + TILs were significantly higher after neoadjuvant therapy (NAT) compared to pre-treatment levels [73]. Patients exhibiting higher post-NAT expression showed better therapeutic responses [73]. Additionally, a closer proximity of tumor cells to CD8 + T cells and dendritic cells was significantly associated with improved response to preoperative chemoradiotherapy (CRT) [74].

Radiation therapy can enhance anti-tumor immunity by facilitating the release and presentation of tumor antigens, thereby promoting CD8 + T cell infiltration into the TME [75]. However, it can also induce the accumulation of immunosuppressive cells, including regulatory T cells, myeloid-derived suppressor cells, and tumor-associated macrophages (TAMs), fostering an immunosuppressive environment and contributing to treatment resistance [75].

Takahashi et al*.* revealed that high stromal PD-L1 expression and increased immune cell infiltration were significantly associated with poor response to neoadjuvant CRT and high tumor budding features in patients with locally advanced rectal cancer (LARC) [76]. Similarly, Liang et al*.* identified that PITPNC1 promotes radiotherapy resistance by reducing CD8 + T cell-mediated immune recognition through the regulation of the FASN/CD155 pathway, thereby inhibiting immune function [77].

The role of TAMs in therapy resistance was highlighted by another study, which found that a higher number of TAMs per high-power field (HPF) independently correlated with poor NAT response [78]. Additionally, CD163 + TAMs were significantly associated with the absence of pCR [78].

#### Hypoxia and Metabolic Reprogramming

Tumor cells adapt to hypoxic conditions by reprogramming metabolism, protein synthesis, and cell cycle progression, primarily through the activation of hypoxia-inducible factors (HIFs) [79]. The HIF-1α subunit drives the overexpression of tumor-associated carbonic anhydrases (CA), particularly CAIX and CAXII, which are often linked to adverse prognostic outcomes in multiple cancers [80–82].

Badon et al*.* investigated CAIX dynamics in rectal cancer and found that NAT significantly increased CAIX expression, with high levels correlating with poor treatment response [83].

#### Angiogenesis and Microvessel Density (MVD)

Microvessel density (MVD), a measure of tumor angiogenesis, has been identified as a predictive factor for treatment outcomes across various cancers [80]. High MVD corresponds to elevated angiogenesis, which, upon radiation, triggers intensified angiogenic signalling and contributes to resistance [84]. MVD was significantly associated with NAT resistance, and high MVD was identified as an independent risk factor for poor outcomes [84].

#### Cancer-Associated Fibroblasts (CAFs)

CAFs play a critical role in shaping the TME by maintaining and remodelling the extracellular matrix [85]. Multiple CAF subsets correlate with prognosis and therapeutic response across various cancers, including melanoma, lung cancer, pancreatic ductal adenocarcinoma, and bladder urothelial cancer [86]. CAFs can survive CRT-induced damage, thereby promoting therapy resistance and tumor progression [87].

Qin et al*.* demonstrated that CAF_FAP enhances epithelial-mesenchymal transition (EMT) via MIR4435-2HG, contributing to poorer chemotherapy outcomes in MSS/pMMR rectal cancer patients [74]. Conversely, CAF_PI16 and CAF_SSLIT2 subsets promote immune cell recruitment and activation, correlating with better therapeutic outcomes [74].

Furthermore, a study identified inflammatory CAFs (iCAFs) as key players in CRT resistance [88]. Upon irradiation, interleukin-1α polarized CAFs toward an inflammatory phenotype and triggered oxidative DNA damage, predisposing them to p53-mediated senescence and resulting in therapy resistance and disease progression [88, 89].

### Microbial Composition and Resistance to Chemoradiotherapy

The gut microbiota, consisting of commensal bacteria and other microorganisms residing on epithelial barriers, not only influences physiological functions but also plays a significant role in the initiation, progression, and dissemination of cancer [90]. Recent studies have demonstrated that gut microbiota can modulate the effectiveness of cancer therapies, including chemoradiotherapy (CRT) [91]. Evidence suggests that individuals with better treatment responses tend to have a gut microbiota profile similar to that of healthy individuals, and that the oral administration of specific gut microbes can enhance the efficacy of chemotherapy [92].

Teng et al*.* conducted a multi-omic profiling study and observed a distinct shift in the fecal microbial community following NAT. Their in vivo and in vitro functional analyses revealed that microbiota-mediated nucleotide synthesis was associated with rectal tumor resistance to NAT [93]. The study cohort was divided into responders and non-responders, with the *Bacteroides *genus, particularly *Bacteroides vulgatus*, becoming selectively enriched in non-responders after NAT regimens [93]. The authors suggested that supplementation with nucleosides or oral administration of *B. vulgatus* protected cancer cells from 5-fluorouracil (5-FU) or radiation treatment [93]. This observation raised the question of whether nucleotide metabolites derived from *B. vulgatus* actively contribute to therapy resistance or whether their presence is a consequence of treatment [94].

Similarly, Sun et al*.* reported significant differences in both alpha and beta diversity of intratumoral microbiota between patients achieving pathological complete response (pCR) and those who did not after NAT [95]. Their study identified 12 microbial species enriched in non-pCR patients, suggesting a negative correlation between these microbial communities and therapeutic response [95].

Jang et al*.* found significant differences in beta diversity, though no differences were observed in alpha diversity between complete responders and non-responders [96]. The study noted that *Bacteroidales* were relatively more abundant in non-CR patients, while the presence of *Duodenibacillus massiliensis* was linked to improved CRT response rates [96].

Shi et al*.* identified notable differences in bacterial taxa between responders and non-responders to NAT [97]. Specifically, *Bacteroides*, *Faecalibacterium/Prausnitzii*, *Clostridium *IV, and *Haemophilus* were enriched in non-responders [97]. The study also suggested that enhanced activity of fatty acid and propanoate metabolism was associated with an improved treatment response, indicating a potential metabolic link between microbiota and therapy outcomes [97].

The gut microbiota significantly influences the response to chemoradiotherapy in rectal cancer. Specific microbial profiles, such as the enrichment of *Bacteroides vulgatus* and other taxa, have been associated with treatment resistance. Conversely, beneficial microbial compositions may enhance therapy response. These findings highlight the potential of microbiota modulation as a therapeutic strategy to improve CRT outcomes in rectal cancer patients. Further research is needed to fully elucidate the causal mechanisms and explore microbiota-targeted interventions.

## Predictive Factors for Response to Neoadjuvant Therapy (NAT)

Table 3 summarizes all the currently known factors that may serve as predictors of response to neoadjuvant therapy (NAT) in rectal cancer. These factors encompass clinical, pathological, molecular, and microbiological variables that have been shown to influence treatment outcomes and guide personalized therapeutic strategies.

**Table 3:** Key factors associated with predicting response to neoadjuvant therapy (NAT) in rectal cancer

Predictor	Type	Correlation	Author	Year
Intratumoral budding	H	HL—PR	Wen et al*.* [98]	2022
Folate receptor alpha	M	HL—PR	Chen et al*.* [99]	2022
CD8 lymphocyte infiltration	IHC	HL—GR	Moghani et al*.* [100]	2021
CD4 + and FoxP3 TILs	IHC	HL—GR	Miyakita et al*.* [101]	2020
Vimentin and tumor-stroma ratio (TSR)	H and IHC	Vimentin low/high TSR- GR	Tian et al*.* [102]	2023
TILS	H + AI	HL—GR	Xu et al*.* [103]	2021
Tumoral stage	I	LL—GR	Georgescu et al*.* [104]	2023
P53 binding protein Immunoscore based on CD3 +/CD8 + cell infiltration	IHC	HL—GR	Huang et al*.* [105]	2019
miR-21	M	HL—PR	Ouro et al*.* [106]	2020
Circulating lymphocytes	C	LL—PR	Lutsyk et al*.* [107]	2023
Circulating cell free DNA	M	LL post-NAT-GR	Truelsen et al*.* [108]	2022
Neutrophil/lymphocyte ratio and platelet/lymphocyte ratio	C	HL—PR	Kim et al*.* [109]	2019
Modified MRI-based split scar sign score	I	LL—GR	Yuan et al*.* [110]	2023
Gross tumor volume GTV	I	GTV < 39.5 cm^3^—GR	Lutsyk et al*.* [111]	2021
Mutant allele tumor heterogeneity score	G	HL—PR	Greenbaum et al*.* [112]	2019
Tumor/stroma ratio	H	Stroma high—PR	Liang et al*.* [113]	2021
MSI +	G	MSI +—PR	Hasan et al*.* [44]	2020
Volumetric sarcopenia	I + AI	volumetric sarcopenia +—PR	Kim et al*.* [114]	2024
Nuclear β catenin	M	HL—PR	Miyako et al*.* [115]	2023

*H* histological, *M* molecular, *IHC* immunohistochemical, *AI* artificial intelligence, *I* imagistic, *C* clinical, *G* genetic, *HL* high levels, *LL* low levels, *PR* poor response, *GR* good response.

## Future Directions in Overcoming Chemoradiotherapy Resistance in Rectal Cancer

Research into chemoradiotherapy (CRT) resistance in rectal cancer is advancing rapidly, with a growing emphasis on understanding the molecular mechanisms of resistance and developing innovative therapeutic strategies.

The discovery of novel molecular biomarkers—spanning genetic, epigenetic, and proteomic markers—holds promise for predicting resistance and optimizing treatment strategies. The development of liquid biopsies offers the potential for real-time monitoring of treatment response, while the integration of radiomics and artificial intelligence (AI) is emerging as a powerful tool for predicting therapeutic outcomes and enhancing personalized treatment planning.

Despite these advancements, significant gaps remain in understanding the tumor microenvironment (TME) and the immune response to CRT. Further research is needed to elucidate the roles of cancer-associated fibroblasts (CAFs), hypoxia, and immune cells in promoting CRT resistance, as well as to explore strategies for modulating the TME to enhance treatment efficacy.

Furthermore, organoid and patient-derived models are becoming powerful tools for overcoming treatment resistance in rectal cancer. They enable personalized therapy, accelerate drug discovery, and improve our understanding of tumor adaptation. Future integration with AI-based predictive modeling, immune profiling, and microbiome studies will further refine their utility in precision oncology.

Genetic research has also accelerated in recent years, focusing on DNA damage response (DDR) and repair mechanisms that influence radiosensitivity. Investigating the role of non-coding RNAs in these processes may provide new avenues for developing targeted therapies. Additionally, personalized treatment strategies based on individual tumor profiling and the identification of novel radiosensitizing agents remain key priorities for future research.

Continued exploration in these areas is essential to overcome CRT resistance and improve long-term outcomes for patients with rectal cancer.

## Conclusion

Chemoradiotherapy (CRT) resistance in rectal cancer remains a significant clinical challenge, driven by a complex interplay of genetic, molecular, cellular, and microenvironmental factors. Despite advancements in neoadjuvant therapy (NAT), many patients show suboptimal responses, with low pathological complete response (pCR) rates and increased recurrence risk.

Addressing CRT resistance requires a multifaceted approach, including investigation of molecular mechanisms, tumor microenvironment dynamics, and gut microbiota influences. Emerging tools such as novel biomarkers, liquid biopsies, predictive models, and AI-integrated radiomics offer promise for real-time monitoring and personalized therapy. Ongoing research into DNA repair pathways, non-coding RNAs, and radiosensitizing strategies is essential. Integrating these insights into clinical practice holds significant potential to overcome CRT resistance and improve patient outcomes.

## Data Availability

No datasets were generated or analysed during the current study.

## Declarations

### Generative AI and AI-Assisted Technologies in the Writing Process

During the preparation of this work, the author(s) used OpenAI. (2025, February 25). ChatGPT (version 4) [large language model]. OpenAI. https://openai.com in order to improve the language and readability. After using this tool/service, the author(s) reviewed and edited the content as needed and take(s) full responsibility for the content of the publication.
